# Differences in tissue-level properties as assessed by nano-scratching in patients with and without atypical femur fractures on long-term bisphosphonate therapy: a proof-of-concept pilot study

**DOI:** 10.1093/jbmrpl/ziae097

**Published:** 2024-07-25

**Authors:** Gabriel Johnson, Lanny V Griffin, Shijing Qiu, Sudhaker D Rao

**Affiliations:** Department of Biomedical Engineering, California Polytechnic State University (Cal Poly), San Luis Obispo, CA, 93407-0365, United States; Department of Biomedical Engineering, California Polytechnic State University (Cal Poly), San Luis Obispo, CA, 93407-0365, United States; Bone & Mineral Research Laboratory, Henry Ford Health/Wayne State University Integrative Biosciences (IBio) Research Facility, Detroit, MI, 48202, United States; Division of Endocrinology, Diabetes, and Bone & Mineral Disorders, Henry Ford Health, Detroit, MI, 48202, United States; Bone & Mineral Research Laboratory, Henry Ford Health/Wayne State University Integrative Biosciences (IBio) Research Facility, Detroit, MI, 48202, United States; Division of Endocrinology, Diabetes, and Bone & Mineral Disorders, Henry Ford Health, Detroit, MI, 48202, United States

**Keywords:** nano-scratch, cortical bone, alendronate, atypical femur fracture, severely suppressed bone turnover

## Abstract

Atypical femur fractures (AFFs) are a well-established complication of long-term bisphosphonate (BP) therapy, but their pathogenesis is not fully understood. Although many patients on long-term BP therapy have severe suppression of bone turnover (SSBT), not all such patients experience AFF, even though SSBT is a major contributor to AFF. Accordingly, we evaluated tissue level properties using nano-scratch testing of trans-iliac bone biopsy specimens in 12 women (6 with and 6 without AFF matched for age and race). Nano-scratch data were analyzed using a mixed-model ANOVA with volume-normalized scratch energy as a function of AFF (Yes or No), region (periosteal or endosteal), and a first-order interaction between region and AFF. Tukey post hoc analyses of the differences of least squared means of scratch energy were performed and reported as significant if *p*<.05. The volume-normalized scratch energy was 10.6% higher in AFF than in non-AFF patients (*p*=.003) and 17.9 % higher in the periosteal than in the endosteal region (*p*=.004). The differences in normalized scratch energy are suggestive of a higher hardness of the bone tissue after long-term BP therapy. The results of this study are consistent with other studies in the literature and demonstrate the efficacy of using Nano-Scratch technique to evaluate bone tissue that exhibits SSBT and AFF. Further studies using nano-scratch may help quantify and elucidate underlying mechanisms for the pathogenesis of AFF.

## Introduction

Atypical femur fractures (AFFs) are a well-documented and serious complication of long-term bisphosphonate or denosumab therapy in patients with osteopenia or osteoporosis.^1–8^ AFFs typically occur along the femoral diaphysis, between the lesser trochanter and the supracondylar flare, through what appears to be a brittle failure with a similar morphology to stress fractures. Prodromal pain is often experienced before AFFs occur, and an increased concentration of linear microcracks (known to degrade bone matrix more severely than other forms of fatigue damage) is found in the presence of AFFs.^9^^,^^10^ However, many factors and features regarding the pathogenesis and severity of AFFs are poorly understood.

Previous studies have shown that duration-dependent severe suppression of bone turnover (SSBT) can alter the properties of cortical tissue in load-bearing long bones.^11–14^ Griffin et al. examined the nanomechanical properties and bone microstructure in patients with and without AFF.^15^ They reported that Wall Thickness, a measure of collective effort of bone multicellular unit designed for bone renewal, was significantly lower in patients with AFF. The indentation modulus and hardness were also found to be greater in patients with an AFF. Additionally, it was also found that cortical bone properties were more affected than cancellous bone properties and that AFF samples displayed significant microcracking and embrittlement, which was consistent with other studies.^16–18^ It was also reported that the benefit of treatment to the risk of an AFF was dependent on the treatment duration and life expectancy of the patient. Therefore, it was concluded that prolonged bisphosphonate therapy (longer than 2 yr) in postmenopausal women was correlated with long-term SSBT that compromised the microstructure of human long-bones containing high amounts of cortical bone and contributes to AFF.^15^

The goal of this study was to evaluate the tissue-level properties of cortical bone in postmenopausal women receiving long-term bisphosphonate therapy for osteoporosis using nano-scratching procedure.^19–21^ In a study by Islam et al., it was reported that the nano-scratch energy per unit volume for genetically modified mice with osteogenesis imperfecta was less than controls.^19^ They suggested that the local failure strength of bone was positively correlated with the energy dissipated by nano-scratching on a volume-normalized basis.^19^ It was therefore hypothesized that the cortical bone of subjects that experienced AFF would require less energy per volume of bone to scratch than that of subjects without an AFF. Additionally, it was hypothesized that there would be a decrease in energy per unit volume, as scratch location moves toward the periosteal surface of cortical bone.

## Materials and methods

### Patient and biopsy selection and sample preparation

The parent study on the prevalence and pathogenesis of AFF consisted of 350 controls who were never exposed to BPs and 451 patients treated with BPs for >2 yr (NCT02155595). Per protocol, we needed 90 biopsies (30 with and 60 without AFF) based on sample size estimate for bone histomorphometry, which was 1 of the 3 specific aims in the parent study (NCT02155595). Of the 80 patients, 20 sustained AFF and 60 did not. A restricted convenient representative sample of 12 osteoporotic, postmenopausal women on long-term alendronate therapy (>2 yr), matched for age, sex, race, BMD, and BMI was selected from a pool of 80 patients who had bone biopsies in the parent study (Table 1). The 12 trans-iliac bone biopsies were embedded in poly(methyl methacrylate) between 2014 and 2018. More detailed information on the subjects, bone histomorphometry, and micro-and nano-indentation results were published recently.^15^^,^^17^^,^^22^

**Table 1:** Demographic and relevant biochemical data of patients with and without an AFF.

**Characteristic**	**Without AFF (*n* = 6)**	**With AFF (*n* = 6)**
**Age (yr)**	68.5 ± 8.46	69.5 ± 10.37
**Alendronate therapy (yr)**	7.33 ± 6.62	13.5 ± 5.23
**Calcium (8.5–10.2 mg/dL)**	9.3 ± 0.16	9.4 ± 0.37
**PTH (15–65 pg/mL)**	39 ± 12	56 ± 18
**25-OHD (>20 ng/mL)**	43 ± 24	54 ± 31
**CTX (104–1008 pg/mL)**	248 ± 98	267 ± 170
**Osteocalcin (8–32 ng/mL)**	15.3 ± 3.35	13.9 ± 4.90
**P1NP (20–108 mcg/L)**	36.7 ± 17.6	25.2 ± 8.67
**Spine BMD (g/cm** ^**2**^ **)**	0.831 ± 0.129	0.878 ± 0.151
**Spine T-Score (SD Units)**	−2.32 ± 0.97	−1.75 ± 1.32
**FN BMD (g/cm** ^**2**^ **)**	0.656 ± 0.100	0.636 ± 0.137
**FN T-Score (SD Units)**	−1.92 ± 0.69	−2.00 ± 1.18

AFF, atypical femur fracture.

All were White women except one Hispanic women in the non-AFF group.

All values are expressed as mean ± SD.

None of the differences was significant most likely due to small sample size and wide variation.

All analyses were performed in the fasting serum sample.

Units of measurements and reference ranges are in parentheses.

### Nano-scratching

**Figure 1:**
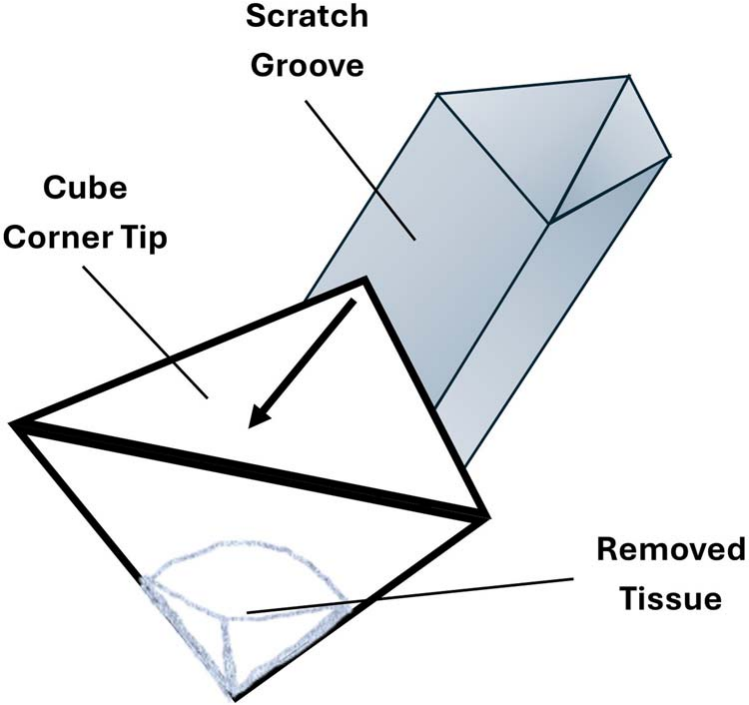
Vector illustration of the cubed-corned scratch head creating a scratch. The scratch illustrated is being dug using the flat of the scratch head. Alternatively, the opposite end could be used to scratch with the edged end of the head.

It is an established microstructural characterization technique that involves moving a sharp tip across the surface of a material (Figure 1). For this study, nano-scratching was performed under a constant load that is perpendicular to the surface, using a diamond cubed-corner indenter tip (*E*_i_ = 1141 GPa, *v*_i_ = 0.07, where *E*_i_ and *v*_i_ are the elastic modulus and Poisson’s ratio of the indenter, respectively). A 50-μm single pass low force topographical scan was performed with the sample moving along the surface with a 0.5-μm/s scanning velocity. The topographic scans were used to provide before-and-after measurements to facilitate measurement of the volume of material removed. A single pass scratch was then performed by applying a 5-mN scratch load after a 5-μm offset for a total scratch length of 45 μm at a scratch velocity of 0.5 μm/s.

**Figure 2:**
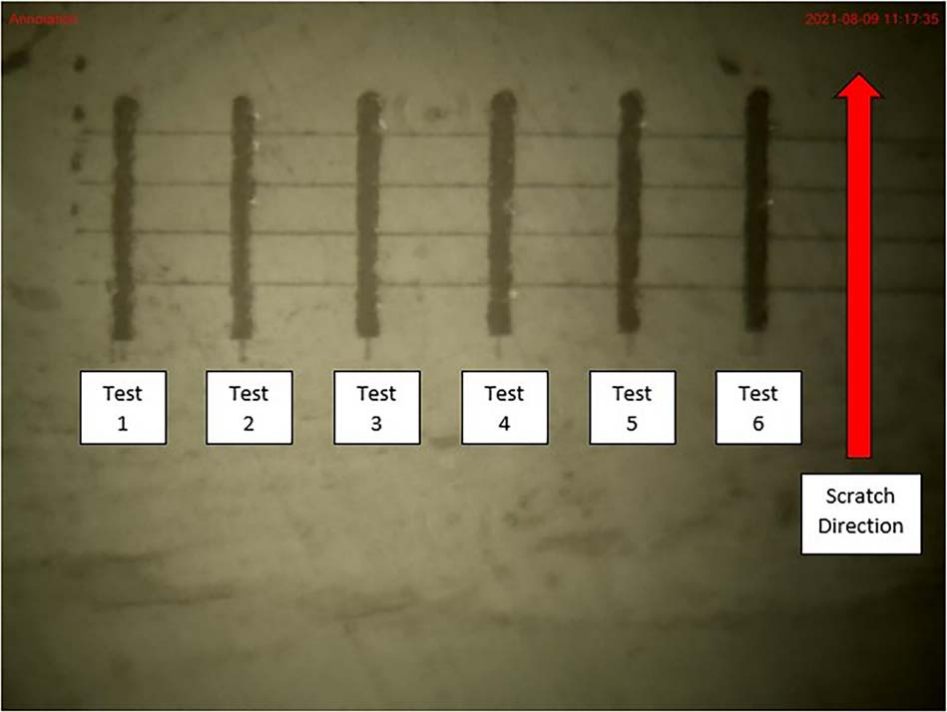
Representative optical microscope image of a set of scratch replicates along the periosteal surface of a bone sample, taken at 20× magnification. The light traces are the topographic scans, whereas the bolder traces are the scratches.

Nano-scratching tests were performed at the endosteal and periosteal surfaces of the biopsies in 6-scratch sets with a 25-μm offset between scratches to minimize the interaction of scratches (Figure 2). A total of 12 endosteal and 12 periosteal scratch replicates were conducted on each sample. The location for a set of 6 scratches was selected based on the region on the cortex (periosteal or endosteal) where the cortical bone was relatively free of natural porosity, such as Haversian or Volkmann canals over the area to be tested. Two sets of 6 scratches were selected, and images were taken at 400× magnification for later visual inspection using the optical microscope on the Nano-indenter (Nanotest 600, Micromaterials Ltd.).

The force along the scratch direction (F_f_) was measured by a friction transducer. Scratch depth (h), transverse distance in the scratch direction (x), and load applied by the indenter (F_n_) were recorded by the Nanotest data acquisition system. The total volume of each scratch (*V*) was calculated by numerically integrating the cross-sectional area (*A*) of the scratch along the scratch direction, *x*.


(1) \begin{equation*} V=\int A\ dx \end{equation*}

The total energy required to produce each scratch (*U_s_*) was calculated by numerically integrating the scratch force (*F_f_*) along the scratch direction. Another view of this parameter is the work required for material removal.


(2) \begin{equation*} {U}_s=\int{F}_f dx \end{equation*}

The volume-normalized scratch energy, *U_v_*, was then calculated by dividing the work of tissue removal (eqn. 2) by the volume of tissue removed (eqn. 1).


(3) \begin{equation*} {U}_v=\frac{U_s}{V} \end{equation*}

Normalizing the work to remove tissue by the volume provides a standardized parameter that allows for comparisons between samples.

### Statistical analyses

A mixed-effects ANOVA was performed with the volume-normalized scratch energy as the dependent variable and the subject as a random factor (SAS 9.4, SAS Institute Inc.). The fixed effects were AFF, the region of the scratch on the bone biopsy (endosteal or periosteal), and an interaction between AFF and region. Post hoc Tukey pairwise differences of the normalized scratch energy and scratch energy for the AFF-region interaction were conducted, with *p*<.05 considered significant.

## Results

The demographic and relevant biochemical data are presented in Table 1. Although none of the differences were significant, AFF patients were slightly older, took alendronate for a much longer period, and had slightly lower bone turnover markers. The lack of difference is almost certainly due to a very small sample size necessitated by available pairs with matching age, ethnicity, type of bisphosphonate, and inevitable wide variation in bone turnover markers. Nevertheless, the trends are in a similar direction as in our previous larger study and all had SSBT on bone biopsy with no difference in histologically determined bone turnover (data not shown).

**Figure 3:**
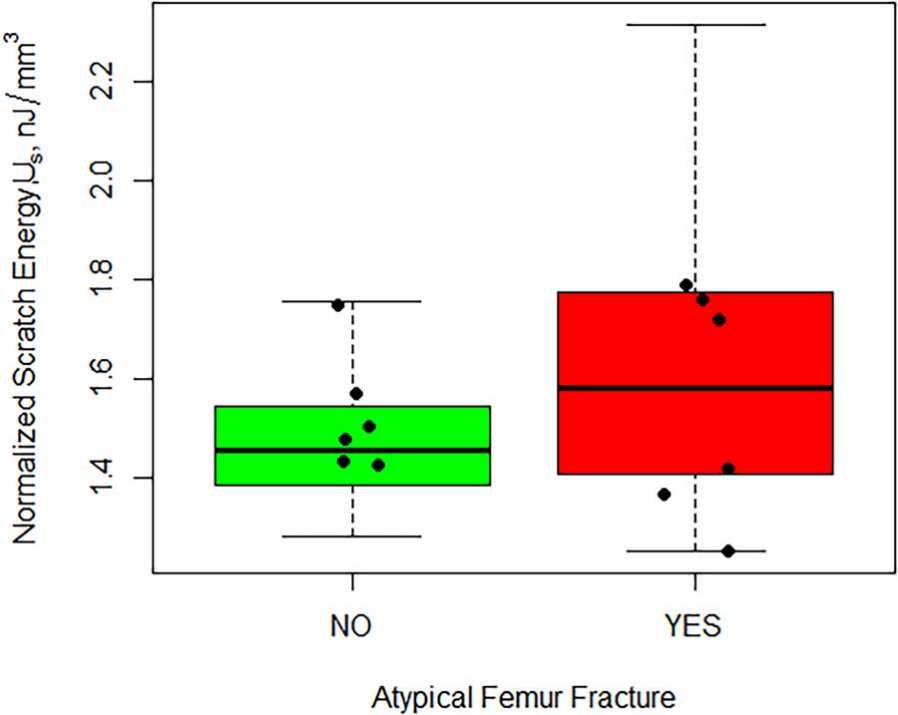
Main effects plot for normalized scratch energy (J/mm^3^).

**Figure 4:**
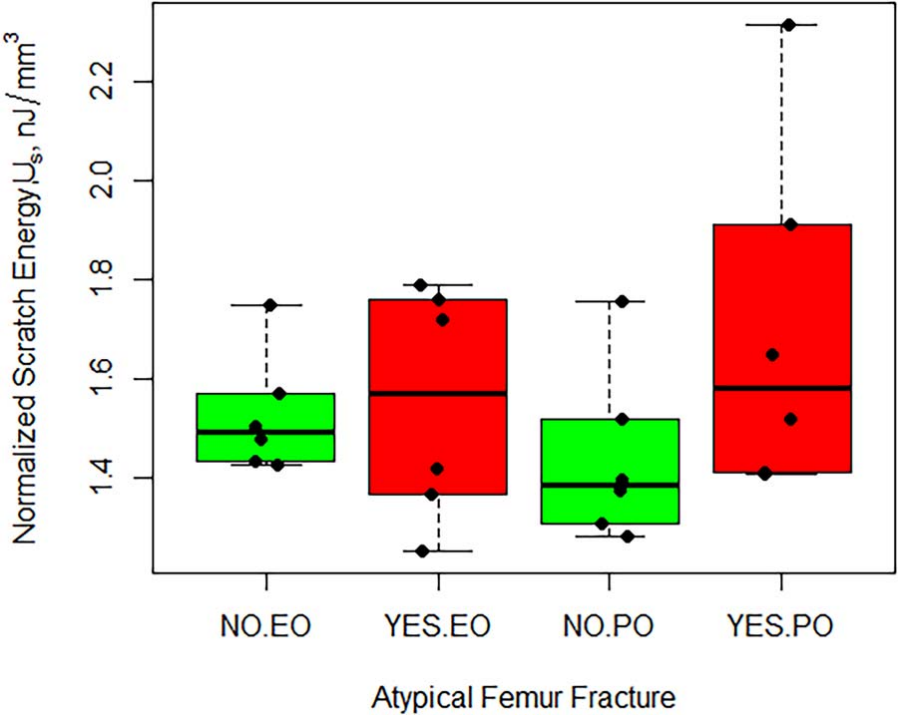
Normalized scratch energy (J/mm^3^) by Region × AFF interaction. Abbreviations: AFF, atypical femur fracture; EO, endosteal; PO, periosteal.

**Figure 5:**
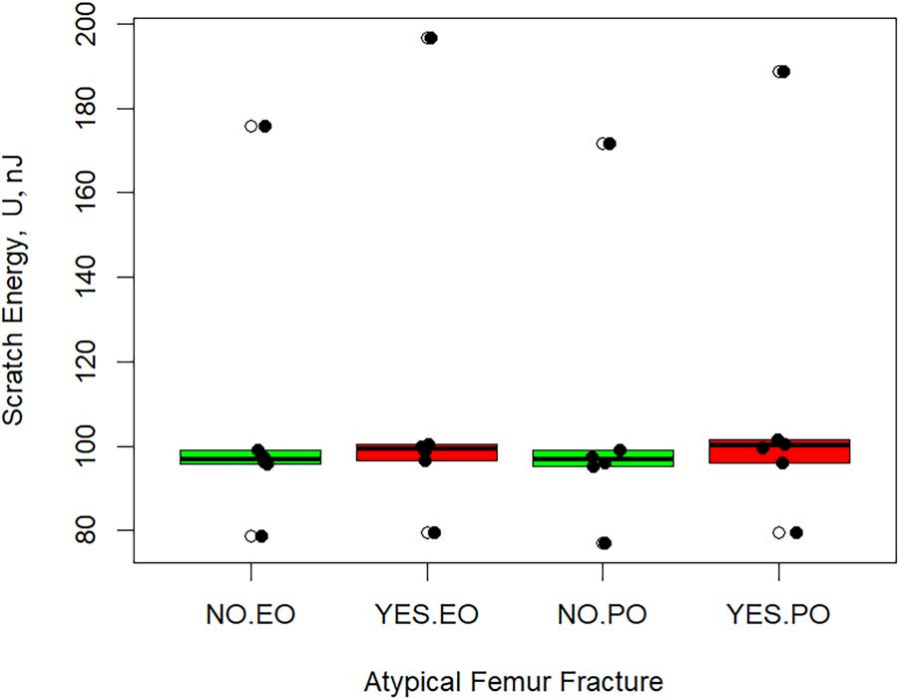
Scratch energy (nJ) by Region × AFF interaction. Abbreviations: AFF, atypical femur fracture; EO, endosteal; PO, periosteal.

The average volume-normalized scratch energy for patients with an AFF was 10.6% higher than in subjects without an AFF although not significant (*p*=.248) (Figure 3). The normalized scratch energy of the region where the scratch was performed (endosteal or periosteal) was likewise not significant (*p*=.341). However, there was a statistically significant interaction between the occurrence of an AFF and scratch location on the normalized scratch energy (Figure 4 and Table 2). When an AFF occurred, the normalized nano-scratch energy in the periosteal region was 11.8% higher than that of the endosteal region (*p*=.0463). In the periosteal region, the average normalized Nano Scratch (NS) energy of subjects with an AFF compared to those without an AFF was 17.3% higher although not significant (*p*=.195). The average normalized nano-scratch energy in the periosteal region of patients with an AFF was 9.70% higher than the endosteal region for subjects without an AFF, although not significant. The energy of tissue removal (not considering the volume of material removed) exhibited similar trends to the volume-normalized scratch energy, although the differences were not significant (Figure 5).

**Table 2:** Tukey pairwise differences of normalized scratch energy.

**Region**	**AFF**	**Region**	**AFF**	**Adj.*p***
**EO**	No	EO	Yes	.997
**EO**	No	PO	No	.557
**EO**	No	PO	Yes	.470
**EO**	Yes	PO	No	.822
**EO**	Yes	PO	Yes	.0463
**PO**	No	PO	Yes	.195

AFF, atypical femur fracture; Adj.*p*, adjusted *p*-value; EO, endosteal; PO, periosteal.

## Discussion

The purpose of this study was to evaluate the NS energy of bone in individuals with SSBT after long-term (>2 yr) bisphosphonate therapy and its relation to AFFs. The 2 major hypotheses of this study were that bone nano-scratch energy would be lower in the samples from patients with an AFF, and that bone nano-scratch energy would decrease near the periosteal cortical bone surface. Both mechanisms were thought to be related to bone tissue toughness as reported by Islam and coworkers.^19^ However, in this study, neither of these hypotheses was confirmed; rather the opposite was found—volume-normalized scratch energy of bone biopsy specimen from AFF patients has a higher volume-normalized scratch energy compared to the bone biopsy specimen from patients without an AFF. This suggests that the mechanisms by which an AFF occurs are not the same as what occurs in an experimental model of osteogenesis imperfecta (OI). For example, it is well known that OI is primarily due to a collagen defect, which affects the collagen/mineral interface.^20^ The pathogenesis of AFF is associated with different factors related to the SSBT, but the mean degree of mineralization and tissue level biomechanical properties are both higher than in normal bone.

**Figure 6:**
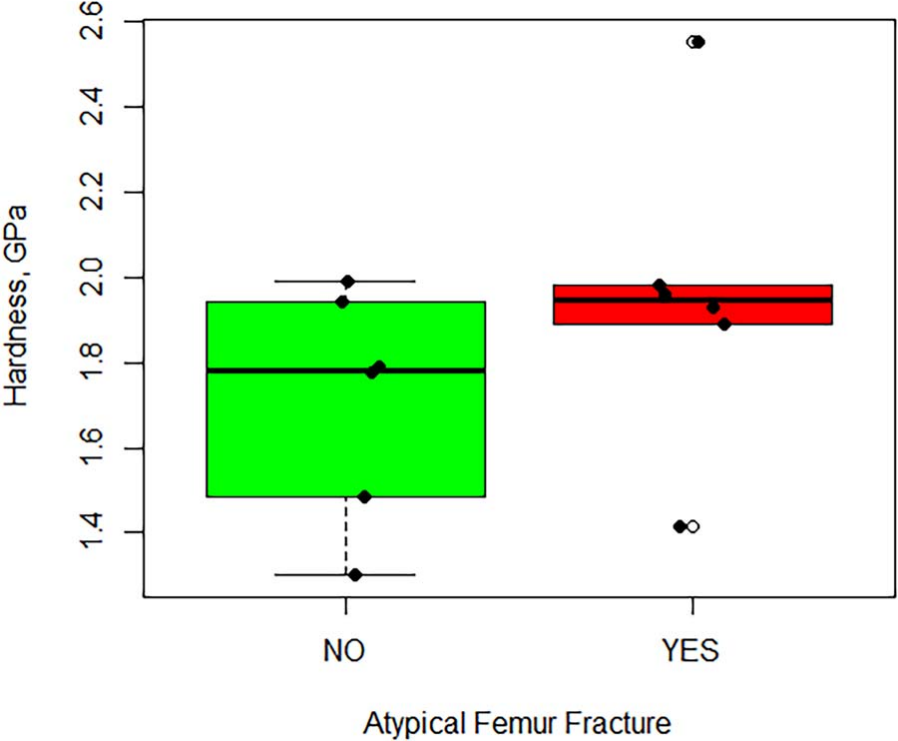
Nanoindentation hardness data for the subset of cortical bone samples used in this study (from Griffin et al.^15^).

It is interesting to note that the amount of energy required to scratch the surface of bone with and without an AFF is the same (Figure 5); however, the volume of tissue removed on the endosteal surface is greater than that of the periosteal surface. Because the volume-normalized energy is calculated by dividing the scratch energy by the volume of the tissue removed (eqn. 3), suggesting that the tissue on the periosteal surface has a higher hardness than the endosteal surface since the probe is not penetrating as deeply, leading to a smaller volume of removed tissue. Materials with a higher hardness are known to have a higher scratch resistance due to the positive correlation with shear strength. An explanation for this is related to the level of mineralization of bone that exhibits SSBT. Farlay et al. recently reported that patients with an AFF had 2.9% higher mean degree of mineralization of bone when compared to controls.^17^ In addition, Griffin et al. reported that hardness was greater in patients with an AFF compared to controls (Figure 6).^15^ Therefore, the finding that bone from patients with an AFF has a greater volume-normalized scratch energy than that of bone without an AFF is consistent with other reports.^15^^,^^17^^,^^21^

In this study, the interactions between AFF and the femoral bone region are significant—especially in the periosteal region. What this suggests is that when an AFF occurs, there are differences in tissue-level properties in the periosteal region that create conditions that increase the likelihood of an AFF. These differences may be associated with a decrease in compositional heterogeneity of the tissue, which has been shown to adversely affect the fracture properties of bone with SSBT.^21^ Furthermore, there is evidence that collagenous changes occur in patients with AFF through increased advanced glycation end products, which tend to lessen the toughness of bone.^23^ Further investigations are needed to elucidate how microstructural changes affect the observed differences in NS energy and their relation to AFF pathogenesis.

A major limitation of this study is the small sample size because this is a pilot study. However, although only 12 samples were examined using the nano-scratch procedure, it is encouraging to note that the test is quite sensitive and demonstrates the efficacy of using nano-scratching method to evaluate the influences of SSBT on bone microstructure, degree of mineralization of bone, and AFF. For example, regional variation was explored in this study. It was shown that there are differences in nano-scratch energy between periosteal and endosteal surfaces. This suggests that further studies using nano-scratch testing combined with other characterization techniques may help elucidate underlying mechanisms of AFF pathogenesis.

## Conclusions

The volume-normalized scratch energy is different in bone with an AFF and occurs primarily in the periosteal region of cortical bone. The difference in normalized scratch energy between the groups with and without an AFF suggests an increased hardness of the tissue, which is consistent with other studies in the literature. Further studies are needed to elucidate the underlying causes of why these microstructural changes occur and how to develop strategies that may mitigate the serious complication of AFF.

## Data Availability

Available upon reasonable request.
